# Assessing the prevalence of *Staphylococcus aureus* in infertile male patients in Tabriz, northwest Iran

**Published:** 2018-07

**Authors:** Aylin Esmailkhani, Mohammad Taghi Akhi, Javid Sadeghi, Behrooz Niknafs, Abed Zahedi Bialvaei, Laya Farzadi, Nooshafarin Safadel

**Affiliations:** 1 *Immunology Research Center, Tabriz University of Medical Sciences, Tabriz, Iran.*; 2 *Department of Bacteriology and Virology, School of Medicine, Tabriz University of Medical Sciences, Tabriz, Iran.*; 3 *Department of Anatomical Sciences, Medical Faculty, Tabriz University of Medical Sciences, Tabriz, Iran.*; 4 *Department of Microbiology, School of Medicine, Iran University of Medical Sciences, Tehran, Iran.*; 5 *Women’s Reproductive Health Research Center, Tabriz University of Medical Sciences, Tabriz, Iran.*; 6 *Department of Quality Management and Laboratory Accreditation, Reference Health Laboratories Research Center, Ministry of Health and Medical Education, Tehran, Iran.*

**Keywords:** Infertility, Staphylococcus aureus, Seminal fluid, MecA

## Abstract

**Background:**

*Staphylococcus aureus* is an infrequent, but one of the most successful bacteria that associated with infertility and are able to spermatozoa immobilization and agglutination.

**Objective:**

The aim of present study was to determine the frequency of *S. aureus* in semen obtained from infertile male patients in northwest Iran.

**Materials and Methods:**

Seminal fluids of 100 infertile men were evaluated. Standard semen examination was done according to World Health Organization guidelines. After isolation and identification of *S. aureus* isolates according to reference methods, determination of susceptibility against important antibiotics and polymerase chain reaction were performed to identify *mecA* and *tst* genes.

**Results:**

Data obtained from the present study shows that 16% of infertile male patients were colonized by *S. aureus*. Ten (62.5%) of the individuals had abnormal seminal fluid sperm motility and morphology and three (18.8%) of them had an abnormal seminal fluid density, whereas after washing with albumin-saline declined to 5 (31.3%), 4 (25%) and 1 (6.3%), respectively. The antibiogram results showed that, except penicillin, other antibiotics have high activity on isolates. Regarding polymerase chain reaction results, *mecA* sequences were detected in 3 (18.7%) strains, whilst the *tst* gene encoding TSST-1 was not detected in any of clinical strains.

**Conclusion:**

It would appear that the *S. aureus* may be an additional negative factor worsening sperm quality and affecting male fertility. Therefore, it demands that all the patients attending in infertility treatment facilities be investigated thoroughly.

## Introduction

Infertility is a subject of worldwide importance and interest in clinical research and practice, which influenced both men and women in reproductive ages (1). Male infertility refers to the inability to achieve spontaneous pregnancy in a fertile female in 1 yr (2). A decline in the male fertility may be attributed to many factors, which include testicular failure, urogenital abnormalities, immunologic problems, varicocele, genetic abnormalities, endocrine disturbances, systemic diseases, cancer, genital tract infections, environmental agents, distorted lifestyle and gonadotoxic factor exposure (2). Different regions of the male reproductive tract are affected by the Infections, such as the epididymis, testis, and male accessory sex glands. Spermatozoa themselves thereafter can be influenced by urogenital infections at various levels of their maturation, development, and transport. Several types of research have shown various types of organisms in semen specimens relying on the detection techniques. However, there is a contradiction with regards to the impact of certain microbial contamination on male infertility. 

It was stated that bacterial detection in the seminal fluid does not necessarily suggest infection, because bacterial isolates in semen may indicate contamination, infection or colonization of the urethral orifice (3). Various bacteria such as *Staphylococcus aureus*, *Ureaplasma urealyticum*, *Mycoplasma* *hominis*, *Chlamydia* *trachomatis*, and *Escherichia* *coli* have been known to impede sperm function in vitro, of which, *S. aureus* has been observed as the causative organism of seminal fluid infections (4). Therefore, microbiology examination of male partners in an infertile couple can be helpful to recognize the cause of male infertility, particularly asymptomatic infections. 

In this study, we aimed to explore the prevalence of *S. aureus* infection in male infertility in Tabriz, northwest Iran. 

## Materials and methods

**Sampling**

The present cross-sectional study was done at the Tabriz University of Medical Sciences, Iran, between July 2015 and April 2016. Seminal fluids of 100 men suffering from unexplained infertility attending to the Department of Obstetrics and Gynecology of Milad Infertility Center were collected either by self or assisted masturbation, after a 3-day abstinence period. 

Before collecting the sample, patients must wash their hands and genital zone with water and soap. Patients should not use any antibiotic for a week before collecting a semen sample. Samples were collected in sterile plastic containers utilized for collecting of urine sample and submitted to the microbiology laboratory within 1 hr of production. To enumeration of sperm cells morphology and sperm count, Nigrosin-Eosin staining method and sodium bicarbonate formalin fluid diluted 1/20 were used, respectively. Control semen of fertile male patients that was obtained from the same clinic also utilized according to the World Health Organization guidelines on semen evaluation and examination. 

Albumin was also required for the development of fertilizing ability by sperm. Therefore, the spermatozoa were washed 2 times with albumin-saline by centrifugation (350 g for 4 min each) and re-suspended in fresh albumin-saline (5). Information’s about the social demographic of every male patient were noted on the form of a structured questionnaire in this study. The maximum and minimum ages of patients who enrolled in this study were 52 and 22 yr, respectively. Patients with infertility due to unrelated reasons with *S. aureus* infections, such as lack of sperm due to corticosteroid treatment were excluded from the study. 

**Isolation and identification**

Standard bacterial culture method (on blood, chocolate and mannitol salt agar) was used to identify microbial agents. Cultures were incubated at 37^o^C for 24-48 hr. After overnight incubation, the isolates were examined by Gram staining technique using catalase, DNase, and coagulase tests, as indicated by microbiology references (6).

**Antibiotic susceptibility testing**

The antimicrobial susceptibility of the *S. aureus* strains was determined in vitro, utilizing the disc diffusion method. The tested antibiotics included penicillin, gentamicin, ciprofloxacin, vancomycin, trimethoprim /sulfamethoxazole and cefoxitin (MAST Diagnostics, Merseyside, UK).

**Detection of** ***mecA*** **and** ***tst*** **genes**

Bacterial DNA was extracted from the isolates according to the tissue buffer boiling method (7). DNA isolates with the concentration of 0.1 ng/µl were used as the templates for analysis. Multiplex PCR was done by CINNAGEN MasterMix (Cinnaclon, Tehran, Iran), which was carried out using the *mecA* and *tst* primers, as described previously (8). The sequences of the *mecA* primers used were 5´-ACTGCTATCCACCCTCAAAC-3´ and 5´-CTGGTGAAGTTGTAATCTGG-3´ and also the sequences of the *tst* primers utilized were 5´-ACCCCTGTTCCCTTATCATC-3´ and 5´-TTTTCAGTATTTGTAACGCC-3´ (synthesized at the Cinnaclon, Tehran, Iran). Amplification was carried out in an Eppendorf Mastercycler thermocycler (Eppendorf, Hamburg, Germany) as follows: 35 cycles of 2 min for denaturation at 94^o^C, 2 min for annealing at 57^o^C, and 1 min for primer extension at 72^o^C, followed by terminal extension at 72^o^C for 7 min. Initial denaturation at 94^o^C for 5 min was used for amplification. Electrophoresis of PCR products was conducted on 1% agarose gel. The gel staining was performed in ethidium bromide for 20 min and visualized in the gel documentation system (UVP, USA). The strains 95-S-739 (*mecA*) and 92-S-1344 (TSST-1) were used as positive controls in this study.

**Ethical consideration**

This research was approved by the ethical committee of regional Medical Research of Tabriz University of Medical Sciences and all patients provided written informed consent for this study (Number: 5/4/13105).

**Statistical analysis**

Statistical analysis was performed with SPSS software (Statistical Package for the Social Sciences, version 19.0, Chicago, IL, USA). The variables were analyzed by univariate analysis using v2 or Fisher's exact test, as appropriate.

## Results

During the study period, a total of 100 infertile male patients were enrolled for the study. Sixteen (16%) of infertile male patients were colonized by *S. aureus* and seven (7%) were colonized by fungi, which were identified by mycology techniques, of which one (6.3%) patients were infected with fungi and S*. aureus*, simultaneously. In the further investigations, we found that all species belonged to the Candida spp. The average age of those infected patients was 35.63±7.31 yr, ranging between 27 and 52 yr. Of the patients, 4 (25%) were under and 11 (68.7%) were over 30 yr of age. Data obtained from the present study shows that 10 (62.5%) of the individuals had abnormal seminal fluid morphology and sperm motility and 3 (18.8%) of the patients had abnormal seminal fluid density whereas after washing with albumin-saline declined to 5 (31.3%), 4 (25%) and 1 (6.3%), respectively (Table I). 

All the other parameters such as pH, viscosity, volume, and liquefaction were found to be normal. In examining the wives of these patients, the endocervix samples of three (15.38%) couples were positive for *S. aureus*. Of that *Candida* spp. carriers also three (3 of 7) wives were infected. The results of antibiotic susceptibility testing are shown in table II. In general, except penicillin, other antibiotics showed high activity on isolates. Regarding PCR results, *mecA* sequences were detected in 3 (18.7%) strains, whilst the *tst* gene encoding TSST-1 was not detected in any of the clinical strains.

**Table I:** Semen profile of the infertile male patients with urogenital tract infection

**Parameter**	**Prior washing**			**After washing**		
**Rating**	**Morphology**	**Motility**	**Density**	**Morphology**	**Motility**	**Density**
Very weak	1 (6.3%)	1 (6.3%)	0 (0%)	0 (0%)	0 (0%)	0 (0%)
Weak	9 (56.3%)	9 (56.3%)	3 (18.8%)	5 (31.3%)	4 (25%)	1 (6.3%)
Intermediate	4 (25%)	4 (25%)	11 (68.8%)	6 (37.5%)	7 (43.8%)	6 (37.5%)
Good	2 (12.5%)	2 (12.5%)	2 (12.5%)	4 (25%)	3 (18.8%)	7 (43.8%)
Excellent	0 (0%)	0 (0%)	0 (0%)	1 (6.3%)	2 (12.5%)	2 (12.5%)
p-value	0.023	0.023	0.010	0.000	0.000	0.090

Data presented as n (%)

**Table II:** Antibiotic susceptibility profile of *Staphylococcus aureus* isolates

**Antibiotic**	**Sensitive**	**Resistant**
Penicillin	4 (25)	12 (75)
Gentamicin	13 (81.3)	3 (18.8)
Ciprofloxacin	13 (81.3)	3 (18.8)
Vancomycin	16 (100)	0 (0)
Co-trimoxazole	15 (93.8)	1 (6.3)
Cefoxitin	13 (81.3)	3 (18.8)

Data presented as n (%)

## Discussion

Bacterial infections are proposed to be significant etiological factors for male infertility. However, despite extended diagnostic efforts providing highly sensitive and specific methods for detection of most of the diseases, the causal link between infection and male infertility has not yet been established. In this study, 16 bacterial growth with *S. aureus* were yielded from a total number of 100 semen samples of infertile males, which this rate is consistent with previous studies (9-11). Ibadin and Ibeh found that an infertile male with infection caused by *S. aureus* has a main role in the deterioration of spermatogenesis and disability of sperm function (9). 

However, other similar studies conducted by other researchers found higher bacterial growth rate than our study. In a study, Orji and their colleagues found that *S. aureus* was the highest prevalent bacteria isolated (37.1%) (12). Rehewy and their colleagues obtained 73% positive bacterial cultures, which the most common aerobic organisms grown were Corynebacterium, *S. aureus*, *S. epidermidis*, *E. coli*, *P. mirabilis*, *K. pneumonia* and Mycoplasma (13). Ekhaise and their co-workers, found that *S. aureus* 7 (77.8%) was the most predominant isolate (14). Also, it was reported that *S. aureus* was the overwhelming flora in infertile male patients with a remarkable decrease in sperm motility (15).

Analysis of etiology has been based on conventional semen profile with information analyzed on the concentration of spermatozoa, the volume of the ejaculate, morphological appearance, their motility, viability and inter-ejaculation variability (10). According to the data, opportunistic microorganisms cause subclinical reproductive tract infections and classical infections of the urogenital tract (16). These seminal fluid infections lead to the suppression of their motility, a decrease in the number of spermatozoa, changes in their morphology and fertilizing capacity. Infectious procedures may lead to impairment of sperm function, deterioration of spermatogenesis, and/or obstruction of the seminal tract. To investigate the impact of *S. aureus* from infertile men on sperm motility, Li and their co-workers collected 60 strains of non-repeated *S. aureus* from the semen of 589 infertile males and analyzed the influence of *S. aureus* on sperm motility using the computer-aided sperm analysis system (17). 

In their study, sperm motility was significantly decreased in 17 of the 60 strains of *S. aureus* (p<0.05). Fortunately, all of the *S. aureus* strains in our study was susceptible to vancomycin, however, we found that the most antibiotic resistance is against penicillin. In a study, the antibiotic susceptibility pattern of Gram positive organisms isolated from 347 semen specimens, *S. aureus* was found 81.83% sensitive to nitrofurantoin followed by levofloxacin (63.6%), gentamycin (54.5%) and Co-trimoxazole (36%) (11). In our study, all isolates that were positive for *mecA* genes were resistant to the gentamicin, ciprofloxacin, and cefoxitin (p<0.05). It was also showed that bacterial detection in semen does not really suggest infection, since bacteria isolates in seminal fluid might represent colonization, contamination or infection of the urethral orifice (4).

Most experts dismiss this infection as insignificance contamination which is thought to be of not important. Semen that goes through the genital tract is ordinarily contaminated with Gram-positive cocci such as Staphylococcus, Streptococcus, Diphteroids and Enterococci (3). Regularely, Enterococci are found in the infertile men semen, and their presence is related to impaired seminal parameters; Staphylococci and Streptococci likewise are regularly present in the urethra of infertile male patients, however unlike Enterococci, do not seem to impair the quality of semen. The most frequently isolated bacteria in industrialized nations are *Ureaplasma urealyticum*, Enterobacteria, and *Chlamydia trachomatis* Every one of these genera seem to be associated with the genesis of epididymitis and prostatitis and therefore may impair fertility (18). 

The results of infertility assessment obtained from questionnaire show that most of the semen samples from these infertile men belong to 37-41 yr old men. Commonly the risk of infertility increases by age, however, the greater part of our investigation patients were under 40 yr. It should be noticed that presence of inflammation and Urogenital Tract Infection posed a threat to the fertility profile of the male patient and should be eradicated by anti-inflammatory treatment and antibiotics, especially during Assisted Reproductive Technique (ART) (19). 

This is because genital bacteria can attach to sperms and some of them can't be expelled even among the sperm washing process in the In vitro Fertilization (IVF) Lab. The most predominant organisms isolated in this system is fungi and *E. coli*. Microbial contamination of the IVF culture media may prompt to fertilization failure (19). Therefore, because of the significant role of bacteriospermia in infertility, more consideration is expected to young men in sexual health.

## Conclusion

It is concluded that, regarding our data, the prevalence of abnormal sperm cells indices and bacterial infection due to *S. aureus* is high. In the management of male factor infertility, *S. aureus* should no longer ignore and must be properly treated.
